# Cognitive Interviewing to Evaluate the Content Validity of a Photo-Supported Preference Assessment

**DOI:** 10.1093/geroni/igab046.1016

**Published:** 2021-12-17

**Authors:** Vanessa Neal, Kelly Knollman-Porter, Rachel Topper, Eleanor McConnell, Katherine Abbott, Kimberly Van Haitsma

**Affiliations:** 1 VA Office of Rural Health, Tampa, Florida, United States; 2 Miami University, Oxford, Ohio, United States; 3 Duke University School of Nursing, Durham, North Carolina, United States; 4 The Pennsylvania State University, University Park, Pennsylvania, United States

## Abstract

Photo-supported verbal assessments have shown to improve comprehension and expression of choices by older adults living with cognitive-communication challenges. The purpose of this study was to assess content validity (CV) of photographs used to supplement the Preferences for Everyday Living Inventory-Nursing Home (PELI-NH) from the perspective of older adults, using cognitive interviewing methods. Participants (N=21) were average age 75 (SD=5.67), mostly male (62%) and white (90%), living in residential communities (86%), with no known cognitive or communication deficits. Interview data was used to iteratively assess and revise photographs. A total of 46 photographs demonstrated CV; 26 demonstrated CV after revisions; 3 did not demonstrate CV after revisions. Content analysis revealed thematic codes describing participants’ photograph preferences including image quality, context, subject diversity, and relevance to long-term care. Discussion will include implications for clinicians and researchers on how to evaluate and improve CV of photo-supported verbal assessments.

